# Synthesis and structure of (*E*)-*N*-(4-meth­oxy­phen­yl)-2-[4-(3-oxo-3-phenyl­prop-1-en-1-yl)phen­oxy]acetamide

**DOI:** 10.1107/S2056989021000864

**Published:** 2021-01-29

**Authors:** Cong Nguyen Tien, Trung Vu Quoc, Dat Nguyen Dang, Giang Le Duc, Luc Van Meervelt

**Affiliations:** a Faculty of Chemistry, Ho Chi Minh City University of Education, 280 An Duong Vuong Street, Ho Chi Minh City, 72711, Vietnam; b Faculty of Chemistry, Hanoi National University of Education, 136 Xuan Thuy, Cau Giay, Hanoi, 11310, Vietnam; c School of Natural Sciences Education, Vinh University, 182 Le Duan Street, Vinh City, 43000, Vietnam; d Department of Chemistry, KU Leuven, Biomolecular Architecture, Celestijnenlaan 200F, Leuven (Heverlee), B-3001, Belgium

**Keywords:** crystal structure, chalcones, hydrogen bonding, C—H⋯π inter­actions, Hirshfeld analysis

## Abstract

The title compound *N*-(4-meth­oxy­phen­yl)-2-[4-(3-oxo-3-phenyl­prop-1-en-1-yl)phen­oxy]acetamide, C_24_H_21_NO_4_, was prepared from reaction of *N*-(4-meth­oxy­phen­yl)-2-chloro­acetamide and (*E*)-3-(4-hy­droxy­phen­yl)-1-phenyl­prop-2-en-1-one, which was obtained from the reaction of 4-hy­droxy­benzaldehyde and aceto­phenone. The structure of the title compound was determined by IR, ^1^H-NMR, ^13^C-NMR and HR–MS spectroscopic data and further characterized by single-crystal X-ray diffraction. The asymmetric unit contains four mol­ecules, each displaying an *E*-configuration of the C=C bond. The dihedral angle between the phenyl rings in each mol­ecule varies between 14.9 (2) and 45.8 (2)°. In the crystal, C—H⋯O hydrogen-bonding inter­actions link the mol­ecules into chains running along the [001] direction. In addition, C—H⋯π inter­actions further stabilize the crystal packing. A Hirshfeld analysis indicates that the most important contributions to the surface contacts are from H⋯H (43.6%), C⋯H/H⋯C (32.1%) and O⋯H/H⋯O (18.1%) inter­actions.

## Abstract 2

The asymmetric unit of the title compound, C_24_H_21_NO_4_, contains four mol­ecules. Each mol­ecule displays intra­molecular N—H⋯O hydrogen bonds and C—H⋯O inter­actions. The crystal packing is characterized by C—H⋯O hydrogen-bonding inter­actions, resulting in chain formation in the [001] direction, and C—H⋯π inter­actions.

## Chemical context   

Chalcones are not only important inter­mediates in the biosynthesis of flavonoids, but are also valuable starting materials for the synthesis of biologically important heterocycles such as pyrazolines, isoxazolines, benzodiazepines and benzo­thia­zepines (Zhuang *et al.*, 2017 ▸; Ovonramwen *et al.*, 2019 ▸). Chalcones and their derivatives have been reported to possess a number of inter­esting biological properties such as anti-inflammatory (Nurkenov *et al.*, 2019 ▸; Vásquez-Martínez *et al.*, 2019 ▸; Hsieh *et al.*, 2000 ▸), anti­cancer (Dimmock *et al.*, 1998 ▸; Bonakdar *et al.*, 2017 ▸; Lim *et al.*, 2020 ▸; Shaik *et al.*, 2020 ▸), anti­oxidant (Ohkatsu & Satoh *et al.*, 2008 ▸; Venkatachalam *et al.*, 2012 ▸; Vásquez-Martínez *et al.*, 2019 ▸; Shaik *et al.*, 2020 ▸), anti­microbial (Fang *et al.*, 2014 ▸; Vásquez-Martínez *et al.*, 2019 ▸; Shaik *et al.*, 2020 ▸) and anti-diabetic activities (Hsieh *et al.*, 2012 ▸; Rammohan *et al.*, 2020 ▸; Konidala *et al.*, 2020 ▸). Besides that, compounds with a phen­oxy-*N*-aryl­acetamide scaffold have demonstrated a variety of biological activities such as anti­microbial (Berest *et al.*, 2011 ▸; Patel *et al.*, 2013 ▸; Williams *et al.*, 2015 ▸), anti­viral (Paramonova *et al.*, 2017 ▸), anti-diabetic (Li *et al.*, 2015 ▸), anti-inflammatory (Rani *et al.*, 2014 ▸), analgesic (Rani *et al.*, 2014 ▸) and anti­cancer (Berest *et al.*, 2011 ▸; Rani *et al.*, 2014 ▸) activities.
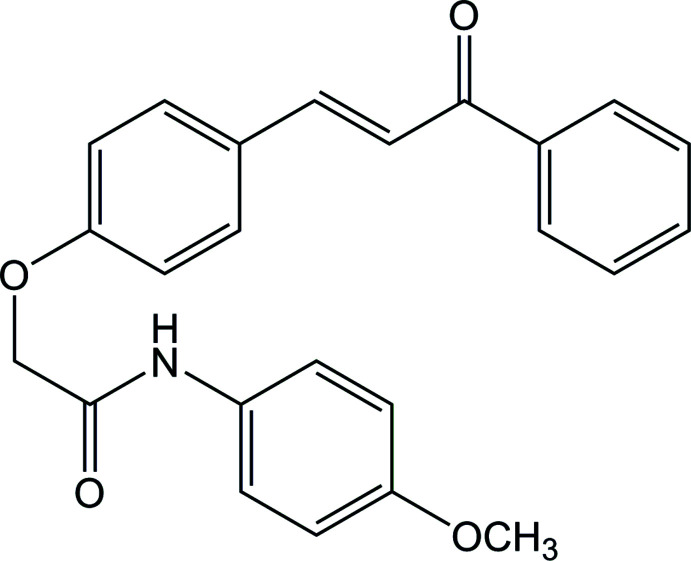


The synthesis of some chalcones containing the phen­oxy-*N*-aryl­acetamide moiety was reported in our previous works and their structures were determined by IR, ^1^H-NMR, ^13^C-NMR and HR-MS spectroscopy (Nguyen *et al.*, 2018 ▸; Bui *et al.*, 2020 ▸). In this work, the synthesis and the mol­ecular and crystal structures of (*E*)-*N*-(4-meth­oxy­phen­yl)-2-[4-(3-oxo-3-phenyl­prop-1-en-1-yl)phen­oxy]acetamide are described in detail.

## Structural commentary   

**Figure 1:**
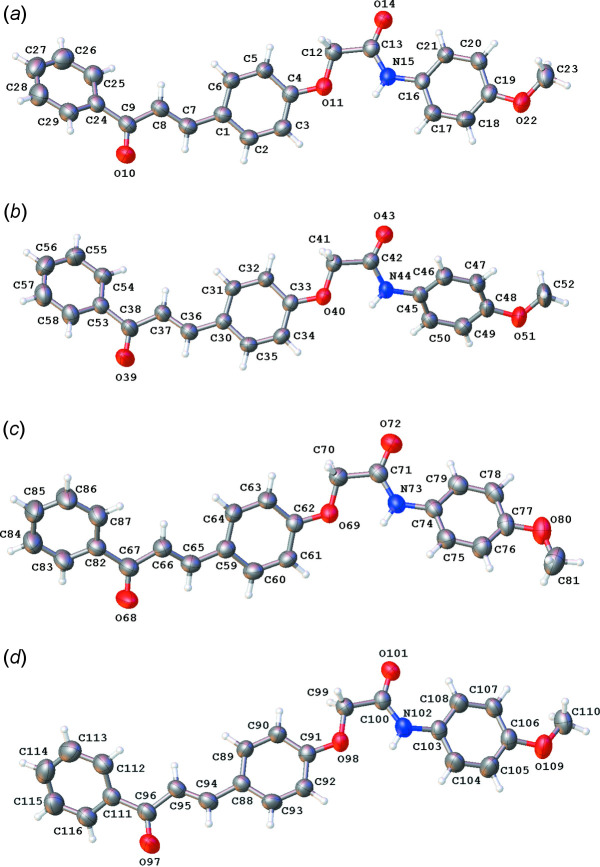
The mol­ecular structure for the four mol­ecules present in the asymmetric unit of the title compound, showing the atom-labelling scheme and displacement ellipsoids at the 50% probability level.

The title compound crystallizes in the monoclinic space group *Cc*. The asymmetric unit contains four mol­ecules and is illustrated in Fig. 1 ▸. In the following discussion, mol­ecule *A* includes atoms C1–C29, mol­ecule *B* atoms C30–C58, mol­ecule *C* atoms C59–C87 and mol­ecule *D* atoms C85–C116. All four mol­ecules exist in the (*E*)-configuration and display intra­molecular N—H⋯O hydrogen bonds and C—H⋯O inter­actions (Table 1 ▸). With the presence of the N—H⋯O hydrogen bond, one would assume the central and the meth­oxy-substituted phenyl rings to be almost coplanar. This is not the case, with dihedral angles between the least-squares planes through the two rings being 17.27 (19), 45.8 (2), 38.91 (19) and 14.9 (2)° for mol­ecules *A*–*D*, respectively. A similar trend is observed for the two phenyl rings linked by the propenone unit, with dihedral angles of 42.8 (2), 29.0 (2), 26.3 (2) and 43.2 (2)° for mol­ecules *A*–*D*, respectively.

**Figure 2:**
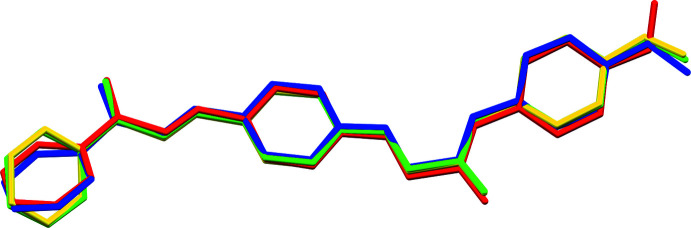
Overlay diagram of the four independent mol­ecules *A* (green), *B* (blue), *C* (red) and *D* (yellow) comprising the asymmetric unit. H atoms are hidden for clarity.

Fig. 2 ▸ shows an overlay diagram of the four mol­ecules *A*–*D* [r.m.s. deviations between 0.0887 Å for the fit of *A* and *D*, and 0.6695 Å for the fit of *C* and *D* as calculated using *Mercury* (Macrae *et al.*, 2020 ▸)]. The largest differences are observed for the terminal groups. At one end, the phenyl rings of mol­ecules *A* and *D*, and of *B* and *C* have a similar orientation. At the other side, the terminal methyl group is oriented differently for mol­ecule *C*.

## Supra­molecular features and Hirshfeld surface analysis   

**Figure 3:**
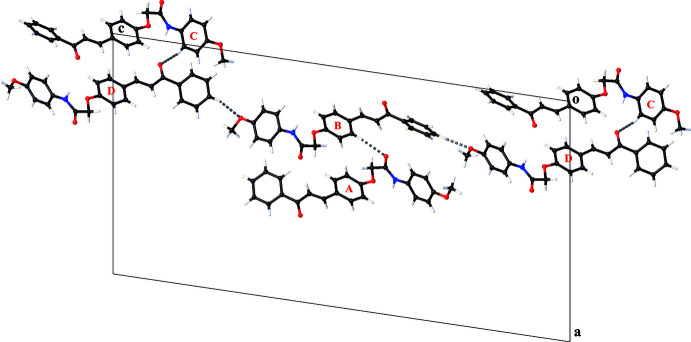
Partial crystal packing of the title compound showing the C—H⋯O inter­actions and chain formation along the [001] direction.

Four C—H⋯O hydrogen bonds are observed between the mol­ecules in the asymmetric unit (Fig. 3 ▸, Table 1 ▸), of which two are involved in a chain formation in the [001] direction through C_arom_—H⋯O_meth­oxy_ inter­actions [graph-set *C*(21)]. In addition, mol­ecules *A* and B, and *C* and *D* inter­act through C_arom_—H⋯O_amide_ inter­actions.

**Figure 4:**
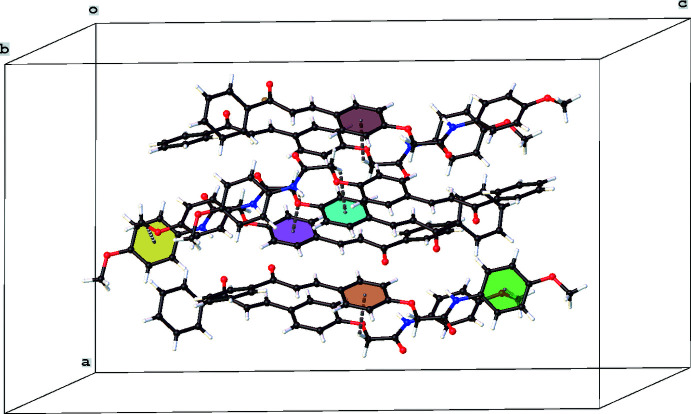
View of the C—H⋯π inter­actions in the crystal packing of the title compound. Colour codes used: cyan for ring C1–C6; orange for ring C30–C35; green for ring C45–C50; magenta for ring C59–C64; yellow for ring C74–C79; brown for ring C88–C93. See Table 1 ▸ for further details.

Despite the presence of many phenyl rings, the crystal packing of the title compound does not show any π–π inter­actions [the shortest inter­centroid distance is 4.754 (2) Å between rings C16–C21 and C74–C79]. However, the crystal packing is mainly characterized by C—H⋯π inter­actions (Fig. 4 ▸, Table 1 ▸). Furthermore, a C=O⋯π inter­action is present in the crystal packing [O43⋯*Cg*9^iv^ = 3.897 (4) Å; *Cg*9 is the centroid of ring C82–C87; symmetry code: (iv) *x*, *y* + 1, *z*]. The packing shows no solvent-accessible voids larger than 15 Å^3^.

**Figure 5:**
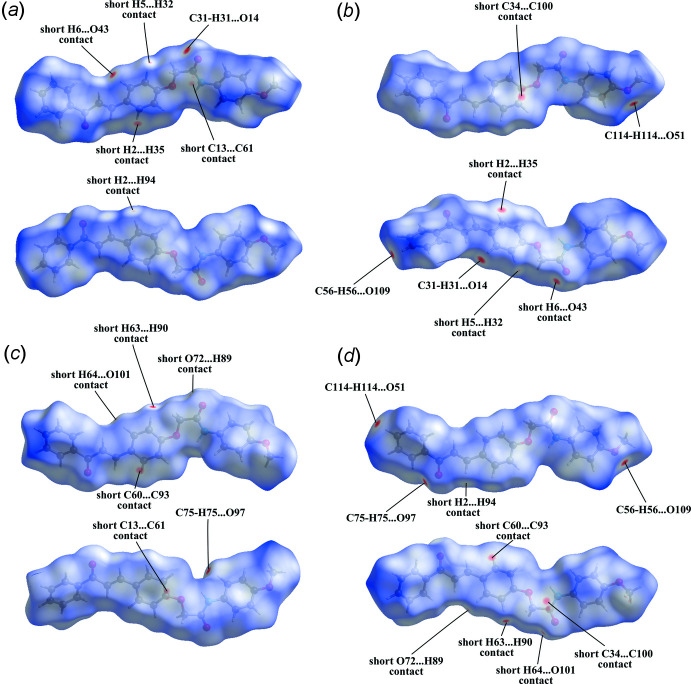
The Hirshfeld surface mapped over *d*_norm_ for the four mol­ecules in the asymmetric unit of the title compound. (*a*) mol­ecule *A* in the range −0.1404 to 1.3398 a.u.; (*b*) mol­ecule *B* in the range −0.1403 to 1.5687 a.u.; (*c*) mol­ecule *C* in the range −0.1240 to 1.8315 a.u.; (*d*) mol­ecule *D* in the range −0.1369 to 1.6590 a.u.

A Hirshfeld surface analysis (Spackman & Jayatilaka, 2009 ▸) and the associated two-dimensional fingerprint plots (McKinnon *et al.*, 2007 ▸) were performed in order to further investigate the supra­molecular network. The Hirshfeld surface calculated using *CrystalExplorer* (Turner *et al.*, 2017 ▸) and mapped over *d*_norm_ is for each mol­ecule in the asymmetric unit given in Fig. 5 ▸. These surfaces show the expected bright-red spots near atoms O14, O51, O97, O109, H31, H56, H75 and H114 involved in the C—H⋯O hydrogen-bonding inter­actions described above. In addition, faint-red spots reveal some additional short H⋯H, C⋯C and H⋯O contacts, as indicated in Fig. 5 ▸. The fingerprint plots indicate that the largest contributions to the Hirshfeld surface come from H⋯H contacts (43.6%) and C⋯H/H⋯C contacts (32.1%), followed by a significant contribution of O⋯H/H⋯O contacts (18.1%). Minor contributions are noted from C⋯O/O⋯C (2.5%), N⋯H/H⋯N (1.5%), C⋯C (1.4%), N⋯C/C⋯N (0.1%) and O⋯N/N⋯O (0.1%) contacts.

## Database survey   

**Figure 6:**
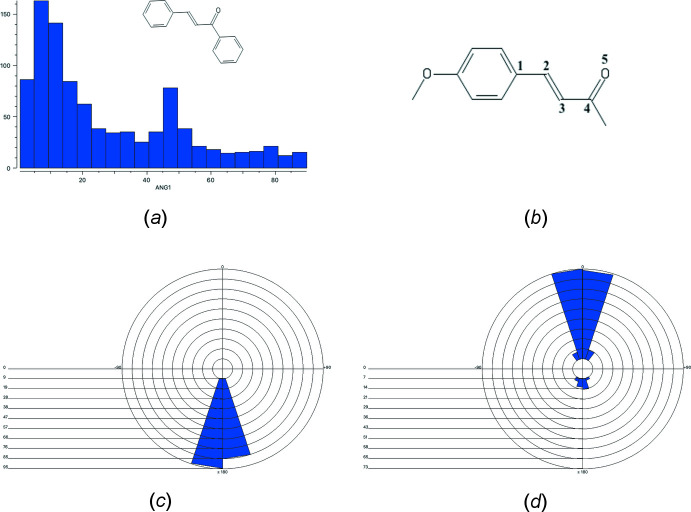
(*a*) Histogram of the dihedral angle between both phenyl rings in chalcones present in the CSD, (*b*) 3-(4-oxyphen­yl)prop-2-en-1-one core of the title compound with numbering used for torsion angles, (*c*) polar histogram of torsion angle 1–2-3–4, (*d*) polar histogram of torsion angle 2–3-4–5.

A search of the Cambridge Structural Database (CSD, Version 5.41, update of May 2020; Groom *et al.*, 2016 ▸) for chalcones (1,3-di­phenyl­prop-2-en-1-one) gave 1168 hits of which 804 have no extra substituents on the prop-2-en-1-one double bond. The histogram of the dihedral angle between the two phenyl rings shows two maxima at ∼15 and ∼55° (Fig. 6 ▸ *a*).

For the 3-(4-oxyphen­yl)prop-2-en-1-one core of the title compound (Fig. 6 ▸ *b*) 159 hits were found. The configuration about the double bond is always *E* with C—C=C—C torsion angles between −168.9 and 169.8° (Fig. 6 ▸ *c*). For the C=C—C=O torsion angle, the majority display an *s-cis* conformation (141 hits or 88.7%), in contrast to an *s-trans* conformation (18 hits, 11.3%) (Fig. 6 ▸ *d*).

In order to verify the frequency of having four mol­ecules in the asymmetric unit, a search in the CSD resulted in only 0.52% of the entries having *Z*′ = 4 (0.62% for *Z*′ ≥ 4).

## Synthesis and crystallization   

**Figure 7:**
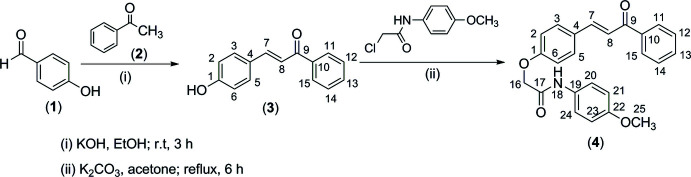
Reaction scheme for the synthesis of the title compound (**4**).

The synthetic pathway to synthesize the target compound, *N*-(4-meth­oxy­phen­yl)-2-[4-(3-oxo-3-phenyl­prop-1-en-1-yl)phen­oxy]acetamide, **4**, is given in Fig. 7 ▸ (numbering on chemical formulae is only used for NMR spectroscopic analysis).

The reaction of 4-hy­droxy­benzaldehyde, **1**, and aceto­phenone, **2**, to obtain chalcone **3** was carried out according to the procedure described in the literature (Dimmock *et al.*, 1998 ▸; Bui *et al.*, 2020 ▸). Physical properties and IR and ^1^H-NMR spectroscopic data of chalcone **3** are in agreement with data in the literature (Dimmock *et al.*, 1998 ▸; Ohkatsu *et al.*, 2008 ▸; Bui *et al.*, 2020 ▸). The existence of chalcone **3** in the (*E*)-configuration is not only clear from the IR spectrum but also the ^1^H-NMR spectrum. While the IR spectrum of **3** shows absorptions at 972 cm^−1^ corresponding to bending vibrations of a *trans*-alkene, its ^1^H-NMR spectrum shows two *doublet* signals (δ 7.73 and 7.75) with a *spin–spin* coupling constant of 17.0 Hz in accordance with a *trans* position.

*N*-(4-meth­oxy­phen­yl)-2-[4-(3-oxo-3-phenyl­prop-1-en-1-yl)phen­oxy]acetamide, **4**, was prepared by stirring a mixture of chalcone **3** and *N*-(4-meth­oxy­phen­yl)-2-chloro­acetamide in acetone containing potassium carbonate. The structure of the product was determined by IR, ^1^H-NMR, ^13^C-NMR and HR–MS spectroscopy.

The mass spectra of **4** showed pseudo-mol­ecular peaks in agreement with the mol­ecular formula of C_24_H_22_NO_4_ (*M*+H)^+^. The IR, ^1^H-NMR and ^13^C-NMR spectra of the product match with the proposed structure. Notably, in the IR spectrum of **4** two new absorption bands appear, one at 3381 (NH) and the other at 1680 cm^−1^ (C=O amide). In comparison to the ^1^H-NMR spectrum of **3**, the spectrum of **4** contains some extra signals in the aromatic area. Moreover, the signal of the CH_2_ group (*singlet* with integration of 2H) is observed at δ 4.77. The *trans* configuration of **4** was also confirmed by the coupling constant *J*_ab_ ≃ 17.0 Hz of the vinylic protons.

*Synthesis of (E)-3-(4-hy­droxy­phen­yl)-1-phenyl­prop-2-en-1-one (**3**):*

To a solution of potassium hydroxide (6 mmol) in 10 mL ethanol, aceto­phenone (2 mmol) was slowly added while stirring for 20 minutes. Then, 4-hy­droxy­benzaldehyde (2 mmol) was continuously added dropwise to the reaction. The mixture was stirred for 3 h at room temperature and kept in a refrigerator overnight. After pouring into ice-cold water, the reaction mixture was acidified with dilute HCl. The solid that separated was filtered, washed thoroughly with water and dried. The crude product was recrystallized from ethanol to afford chalcone **3** (yield 77%) in the form of yellow crystals (m.p. 465–467 K). IR (Shimadzu FTIR-8400S, KBr, cm^−1^): 972 (C=C), 1600 (C=C), 1651 (C=O), 3017 (C—H), 3225 (*broad*, OH); ^1^H NMR [Bruker XL-500, 500 MHz, *d*_6_-DMSO, (ppm), *J* (Hz)]: 6.86 (2H, *d*, *J* = 8.5 Hz, H^2^ and H^6^), 7.57 (2H, *dd*, *J* =7.5 Hz, *J* = 7.0 Hz, H^12^ and H^14^), 7.66 (1H, *dd*, *J* = 6.0 Hz, *J* = 7.0 Hz, H^13^), 7.73 (1H, *d*, *J* = 16.5 Hz, H^8^), 7.74 (1H, *d*, *J* = 17.0 Hz, H^7^), 7.76 (2H, *d*, *J* = 8.0 Hz, H^3,5^), 8.13 (2H, *d*, *J* = 8.0 Hz, H^11^ and H^15^), 10.12 (1H, *s*, OH). ^13^C NMR [Bruker XL-500, 125 MHz, *d*_6_-DMSO, (ppm)]: 115.8 (C^2^ and C^6^), 118.5 (C^8^), 125.8, 128.3, 128.7, 131.1, 132.8, 137.9, 144.5 (C^7^), 160.2 (C^1^), 189.0 (C^9^).

*Synthesis of (E)*-*N-(4-meth­oxy­phen­yl)-2-(4-(3-oxo-3-phenyl­prop-1-en-1-yl)phen­oxy)acetamide (**4**):*

To a solution containing chalcone **3** (1 mmol) dissolved in 10 mL dry acetone potassium carbonate (1.2 mmol) was added. After stirring 20 minutes, a solution of 2-chloro-*N*-(4-meth­oxy­phen­yl)acetamide (1 mmol) in acetone (10 mL) was added dropwise. The reaction mixture was refluxed for 6 h and then cooled to room temperature. After pouring in ice-cold water, the solid separated was filtered and recrystallized from ethanol to obtain **4** (yield 61%) in the form of colourless needle-shaped crystals (m.p. 430-431 K). IR (Shimadzu FTIR-8400S, KBr, cm^−1^): 986 (C=C), 1242, 1064 (C—O—C), 1589, 1543, 1435 (C=C), 1680, 1656 (C=O), 2908 (C*sp* ^3^—H), 3039 (C*sp* ^2^—H), 3381 (N—H); ^1^H NMR [Bruker XL-500, 500 MHz, *d*_6_-DMSO, (ppm), *J* (Hz)]: 3.74 (3H, *s*, H^25^), 4.77 (2H, *s*, H^16^), 6.91 (2H, *d*, *J* = 9.0, H^21^ and H^23^), 7.10 (2H, *d*, *J* = 9.0, H^2^ and H^6^), 7.56 (2H, *d*, *J* = 8.0, H^20^ and H^24^), 7.58 (2H, *dd*, *J* = 7.0, H^12^ and H^14^), 7.67 (1H, *dd*, *J* = 7.0, H^13^), 7.74 (1H, *d*, *J* = 15.5, H^8^), 7.83 (1H, *d*, *J* = 15.5, H^7^), 7.89 (2H, *d*, *J* = 9.0, H^3^ and H^5^), 8.15 (2H, *d*, *J* = 7.5, H^11^ and H^15^), 10.00 (1H, *s*, H^18^). ^13^C NMR [Bruker XL-500, 125 MHz, *d*_6_-DMSO, (ppm)]: 55.7 (C^25^), 67.6 (C^16^), 114.3 (C^2^ and C^6^), 115.6 (C^21^ and C^23^), 120.4 (C^20^ and C^24^), 121.8, 128.4, 128.9, 129.2, 131.2, 131.9, 133.4, 138.3, 144.3, 156.1 (C^22^), 160.4 (C^1^), 166.2 (C^17^), 189.5 (C^9^). Calculation for C_24_H_22_NO_4_ (*M*+H): 388.1549; found: 388.1542 (*M*+H)^+^.

## Refinement   

Crystal data, data collection and structure refinement details are summarized in Table 2 ▸. The H atoms H15, H44, H73 and H102 were located from difference electron-density maps and refined freely [for H44, an N44—H44 distance restraint of 0.87 (2) Å was used]. The other H atoms were placed in idealized positions and included as riding contributions with *U*_iso_(H) values of 1.2*U*_eq_ or 1.5*U*_eq_ of the parent atoms, with C—H distances of 0.93 (aromatic), 0.97 (CH_2_) and 0.96 Å (CH_3_). In the final cycles of refinement, 26 outliers were omitted. Refinement of the Flack parameter [0.1 (3)] did not allow the unambiguous determination of the chirality of the spatial mol­ecular arrangement in space group *Cc*.

## Supplementary Material

Crystal structure: contains datablock(s) I. DOI: 10.1107/S2056989021000864/ey2003sup1.cif

Structure factors: contains datablock(s) I. DOI: 10.1107/S2056989021000864/ey2003Isup2.hkl

CCDC reference: 2058520

Additional supporting information:  crystallographic information; 3D view; checkCIF report

## Figures and Tables

**Table 1:** Hydrogen-bond geometry (Å, °) *Cg*1, *Cg*4, *Cg*5, *Cg*7, *Cg*8 and *Cg*10 are the centroids of the C1–C6, C30–C35, C45–C50, C59–C64, C74–C79 and C88–C93 rings, respectively.

*D*—H⋯*A*	*D*—H	H⋯*A*	*D*⋯*A*	*D*—H⋯*A*
N15—H15⋯O11	0.84 (5)	2.14 (5)	2.602 (4)	115 (4)
N44—H44⋯O40	0.83 (3)	2.11 (3)	2.605 (4)	118 (3)
N73—H73⋯O69	0.86 (4)	2.12 (4)	2.579 (4)	113 (3)
N102—H102⋯O98	0.86 (4)	2.09 (4)	2.603 (4)	118 (3)
C7—H7⋯O10	0.93	2.47	2.807 (5)	101
C21—H21⋯O14	0.93	2.32	2.925 (5)	122
C36—H36⋯O39	0.93	2.47	2.811 (6)	102
C46—H46⋯O43	0.93	2.47	2.964 (5)	113
C65—H65⋯O68	0.93	2.46	2.803 (6)	102
C79—H79⋯O72	0.93	2.48	2.992 (5)	115
C94—H94⋯O97	0.93	2.46	2.804 (6)	102
C108—H108⋯O101	0.93	2.33	2.934 (5)	122
C31—H31⋯O14	0.93	2.57	3.494 (5)	175
C56—H56⋯O109^i^	0.93	2.57	3.487 (6)	167
C75—H75⋯O97^ii^	0.93	2.57	3.409 (6)	151
C114—H114⋯O51^iii^	0.93	2.56	3.444 (7)	159
C12—H12*A*⋯*Cg*7^iv^	0.97	2.81	3.522 (4)	131
C23—H23*A*⋯*Cg*8^iv^	0.96	2.72	3.669 (5)	168
C41—H41*B*⋯*Cg*10^v^	0.97	2.84	3.592 (4)	135
C70—H70*A*⋯*Cg*1	0.97	2.92	3.667 (5)	135
C99—H99*B*⋯*Cg*4^vi^	0.97	2.80	3.517 (4)	131
C110—H11*C*⋯*Cg*5^vi^	0.96	2.64	3.569 (5)	162

Symmetry codes: (i) 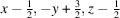; (ii) 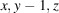; (iii) 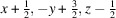; (iv) 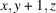; (v) 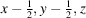; (vi) 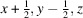.

**Table 2:** Experimental details

Crystal data	
Chemical formula	C_24_H_21_NO_4_
*M*_r_	387.42
Crystal system, space group	Monoclinic, *C* *c*
Temperature (K)	293
*a*, *b*, *c* (Å)	20.3693 (9), 10.0956 (4), 39.1991 (16)
β (°)	98.443 (4)
*V* (Å^3^)	7973.5 (6)
*Z*	16
Radiation type	Mo *K*α
μ (mm^−1^)	0.09
Crystal size (mm)	0.5 × 0.3 × 0.1

Data collection	
Diffractometer	Rigaku Oxford Diffraction SuperNova, Single source at offset/far, Eos
Absorption correction	Multi-scan (*CrysAlis PRO*; Rigaku OD, 2018 ▸)
*T*_min_, *T*_max_	0.726, 1.000
No. of measured, independent and observed [*I* > 2σ(*I*)] reflections	25709, 13137, 10463
*R*_int_	0.015
(sin θ/λ)_max_ (Å^−1^)	0.625

Refinement	
*R*[*F* ^2^ > 2σ(*F* ^2^)], *wR*(*F* ^2^), *S*	0.045, 0.127, 1.01
No. of reflections	13137
No. of parameters	1065
No. of restraints	3
H-atom treatment	H atoms treated by a mixture of independent and constrained refinement
Δρ_max_, Δρ_min_ (e Å^−3^)	0.14, −0.14
Absolute structure	Flack *x* determined using 3066 quotients [(*I* ^+^)−(*I* ^−^)]/[(*I* ^+^)+(*I* ^−^)] (Parsons *et al.*, 2013 ▸)
Absolute structure parameter	0.1 (3)

Computer programs: *CrysAlis PRO* (Rigaku OD, 2018 ▸), *SHELXT2014/5* (Sheldrick, 2015 ▸ ▸), *SHELXL2016/4* (Sheldrick, 2015 ▸ ▸) and *OLEX2* (Dolomanov *et al.*, 2009 ▸).

## supplementary crystallographic information

### Crystal data

**Table:**

C_24_H_21_NO_4_	*F*(000) = 3264
*M**_r_* = 387.42	*D*_x_ = 1.291 Mg m^−^^3^
Monoclinic, *C**c*	Mo *K*α radiation, λ = 0.71073 Å
*a* = 20.3693 (9) Å	Cell parameters from 10524 reflections
*b* = 10.0956 (4) Å	θ = 2.6–26.3°
*c* = 39.1991 (16) Å	µ = 0.09 mm^−^^1^
β = 98.443 (4)°	*T* = 293 K
*V* = 7973.5 (6) Å^3^	Plate, colourless
*Z* = 16	0.5 × 0.3 × 0.1 mm

### Data collection

**Table:**

Rigaku Oxford Diffraction SuperNova, Single source at offset/far, Eos diffractometer	13137 independent reflections
Radiation source: micro-focus sealed X-ray tube, SuperNova (Mo) X-ray Source	10463 reflections with *I* > 2σ(*I*)
Mirror monochromator	*R*_int_ = 0.015
Detector resolution: 15.9631 pixels mm^-1^	θ_max_ = 26.4°, θ_min_ = 2.4°
ω scans	*h* = −25→25
Absorption correction: multi-scan (CrysAlisPro; Rigaku OD, 2018)	*k* = −12→12
*T*_min_ = 0.726, *T*_max_ = 1.000	*l* = −48→39
25709 measured reflections	

### Refinement

**Table:**

Refinement on *F*^2^	Hydrogen site location: mixed
Least-squares matrix: full	H atoms treated by a mixture of independent and constrained refinement
*R*[*F*^2^ > 2σ(*F*^2^)] = 0.045	*w* = 1/[σ^2^(*F*_o_^2^) + (0.0536*P*)^2^ + 3.8381*P*] where *P* = (*F*_o_^2^ + 2*F*_c_^2^)/3
*wR*(*F*^2^) = 0.127	(Δ/σ)_max_ < 0.001
*S* = 1.01	Δρ_max_ = 0.14 e Å^−^^3^
13137 reflections	Δρ_min_ = −0.14 e Å^−^^3^
1065 parameters	Absolute structure: Flack *x* determined using 3066 quotients [(*I*^+^)-(*I*^-^)]/[(*I*^+^)+(*I*^-^)] (Parsons *et al.*, 2013)
3 restraints	Absolute structure parameter: 0.1 (3)
Primary atom site location: dual	

### Special details

**Table:**

Geometry. All esds (except the esd in the dihedral angle between two l.s. planes) are estimated using the full covariance matrix. The cell esds are taken into account individually in the estimation of esds in distances, angles and torsion angles; correlations between esds in cell parameters are only used when they are defined by crystal symmetry. An approximate (isotropic) treatment of cell esds is used for estimating esds involving l.s. planes.

### Fractional atomic coordinates and isotropic or equivalent isotropic displacement parameters (Å^2^)

**Table:**

	*x*	*y*	*z*	*U*_iso_*/*U*_eq_	
C1	0.52538 (18)	0.3310 (4)	0.51031 (10)	0.0526 (9)	
C2	0.54903 (19)	0.3342 (4)	0.47879 (11)	0.0575 (10)	
H2	0.582601	0.275806	0.475129	0.069*	
C3	0.52457 (19)	0.4208 (4)	0.45271 (11)	0.0573 (10)	
H3	0.542349	0.421858	0.432172	0.069*	
C4	0.47359 (18)	0.5058 (4)	0.45736 (10)	0.0502 (9)	
C5	0.4473 (2)	0.5021 (4)	0.48813 (11)	0.0594 (10)	
H5	0.411984	0.557216	0.491147	0.071*	
C6	0.47331 (19)	0.4169 (4)	0.51426 (11)	0.0573 (10)	
H6	0.455802	0.416758	0.534873	0.069*	
C7	0.5573 (2)	0.2467 (4)	0.53826 (12)	0.0587 (10)	
H7	0.591131	0.192774	0.532646	0.070*	
C8	0.5452 (2)	0.2361 (4)	0.57022 (12)	0.0599 (10)	
H8	0.508842	0.280461	0.576615	0.072*	
C9	0.5873 (2)	0.1562 (4)	0.59613 (12)	0.0620 (11)	
O10	0.64060 (17)	0.1112 (4)	0.59019 (9)	0.0906 (11)	
O11	0.45280 (14)	0.5899 (3)	0.43058 (7)	0.0605 (7)	
C12	0.39867 (19)	0.6763 (4)	0.43359 (11)	0.0557 (10)	
H12A	0.407276	0.724061	0.455258	0.067*	
H12B	0.358500	0.624732	0.433597	0.067*	
C13	0.3889 (2)	0.7738 (4)	0.40405 (11)	0.0533 (9)	
O14	0.34232 (14)	0.8522 (3)	0.40251 (8)	0.0680 (8)	
N15	0.43297 (19)	0.7680 (3)	0.38221 (9)	0.0578 (9)	
H15	0.465 (2)	0.713 (5)	0.3875 (14)	0.090 (18)*	
C16	0.44092 (19)	0.8534 (4)	0.35437 (10)	0.0500 (9)	
C17	0.4945 (2)	0.8326 (4)	0.33725 (12)	0.0629 (11)	
H17	0.524200	0.764848	0.344451	0.076*	
C18	0.5046 (2)	0.9100 (5)	0.30983 (13)	0.0727 (13)	
H18	0.541150	0.894121	0.298688	0.087*	
C19	0.4613 (2)	1.0113 (4)	0.29851 (11)	0.0558 (10)	
C20	0.4082 (2)	1.0352 (4)	0.31552 (11)	0.0593 (10)	
H20	0.379305	1.104514	0.308508	0.071*	
C21	0.3976 (2)	0.9561 (4)	0.34316 (11)	0.0586 (10)	
H21	0.361163	0.972090	0.354312	0.070*	
O22	0.47526 (17)	1.0813 (3)	0.27015 (9)	0.0785 (9)	
C23	0.4348 (3)	1.1920 (5)	0.25957 (13)	0.0838 (15)	
H23A	0.437403	1.254665	0.278138	0.126*	
H23B	0.389620	1.163502	0.253399	0.126*	
H23C	0.449795	1.232871	0.240003	0.126*	
C24	0.5659 (2)	0.1334 (4)	0.63042 (11)	0.0569 (10)	
C25	0.4990 (2)	0.1271 (5)	0.63412 (13)	0.0710 (12)	
H25	0.466491	0.145226	0.615464	0.085*	
C26	0.4814 (3)	0.0938 (5)	0.66570 (16)	0.0843 (15)	
H26	0.436651	0.086164	0.667741	0.101*	
C27	0.5276 (3)	0.0719 (5)	0.69399 (15)	0.0863 (15)	
H27	0.514432	0.050205	0.715027	0.104*	
C28	0.5939 (3)	0.0821 (6)	0.69107 (13)	0.0891 (15)	
H28	0.625908	0.069040	0.710285	0.107*	
C29	0.6130 (2)	0.1121 (5)	0.65942 (12)	0.0722 (12)	
H29	0.657875	0.117932	0.657541	0.087*	
C88	0.71540 (18)	0.9620 (4)	0.47620 (10)	0.0510 (9)	
C89	0.7682 (2)	0.8784 (4)	0.47261 (11)	0.0578 (10)	
H89	0.786685	0.880066	0.452281	0.069*	
C90	0.7937 (2)	0.7927 (4)	0.49876 (11)	0.0610 (11)	
H90	0.829296	0.738307	0.495928	0.073*	
C91	0.76653 (19)	0.7872 (4)	0.52917 (10)	0.0520 (9)	
C92	0.71456 (19)	0.8711 (4)	0.53360 (11)	0.0573 (10)	
H92	0.696222	0.869185	0.553964	0.069*	
C93	0.69038 (19)	0.9573 (4)	0.50755 (11)	0.0615 (11)	
H93	0.656147	1.014576	0.510927	0.074*	
C94	0.6835 (2)	1.0468 (4)	0.44841 (12)	0.0611 (11)	
H94	0.649897	1.101436	0.454069	0.073*	
C95	0.6959 (2)	1.0566 (4)	0.41620 (11)	0.0582 (10)	
H95	0.732344	1.012856	0.409788	0.070*	
C96	0.6526 (2)	1.1356 (4)	0.39051 (12)	0.0640 (11)	
O97	0.60033 (18)	1.1850 (4)	0.39709 (9)	0.0937 (11)	
O98	0.78686 (14)	0.7032 (3)	0.55599 (7)	0.0620 (7)	
C99	0.8402 (2)	0.6152 (4)	0.55292 (11)	0.0594 (10)	
H99A	0.880941	0.665389	0.553548	0.071*	
H99B	0.831705	0.569556	0.530948	0.071*	
C100	0.8481 (2)	0.5145 (4)	0.58202 (11)	0.0545 (9)	
O101	0.89334 (14)	0.4346 (3)	0.58359 (8)	0.0686 (8)	
N102	0.80389 (19)	0.5237 (3)	0.60418 (9)	0.0611 (9)	
H102	0.774 (2)	0.583 (4)	0.5981 (11)	0.059 (12)*	
C103	0.7953 (2)	0.4403 (4)	0.63206 (10)	0.0543 (9)	
C104	0.7429 (2)	0.4676 (5)	0.64987 (13)	0.0754 (13)	
H104	0.714696	0.537950	0.642796	0.090*	
C105	0.7317 (3)	0.3927 (5)	0.67778 (14)	0.0834 (15)	
H105	0.696575	0.413396	0.689564	0.100*	
C106	0.7729 (2)	0.2864 (4)	0.68831 (11)	0.0619 (10)	
C107	0.8236 (2)	0.2568 (4)	0.67065 (11)	0.0642 (11)	
H107	0.850896	0.184672	0.677401	0.077*	
C108	0.8353 (2)	0.3330 (4)	0.64263 (12)	0.0688 (12)	
H108	0.870313	0.311638	0.630873	0.083*	
O109	0.75760 (18)	0.2193 (3)	0.71677 (9)	0.0859 (10)	
C110	0.7891 (3)	0.0976 (5)	0.72520 (14)	0.0794 (14)	
H11A	0.771350	0.058445	0.744225	0.119*	
H11B	0.835878	0.111699	0.731508	0.119*	
H11C	0.781475	0.039459	0.705648	0.119*	
C111	0.6718 (2)	1.1522 (4)	0.35509 (12)	0.0570 (10)	
C112	0.7367 (2)	1.1578 (5)	0.34995 (14)	0.0754 (13)	
H112	0.770263	1.145380	0.368483	0.090*	
C113	0.7530 (3)	1.1818 (5)	0.31752 (17)	0.0925 (17)	
H113	0.797270	1.189023	0.314471	0.111*	
C114	0.7033 (3)	1.1950 (5)	0.28952 (16)	0.0919 (17)	
H114	0.713993	1.210841	0.267622	0.110*	
C115	0.6381 (3)	1.1846 (5)	0.29442 (14)	0.0869 (15)	
H115	0.604606	1.190101	0.275623	0.104*	
C116	0.6221 (2)	1.1661 (5)	0.32712 (13)	0.0715 (12)	
H116	0.577831	1.162845	0.330387	0.086*	
C30	0.21747 (18)	0.9533 (4)	0.48202 (10)	0.0496 (9)	
C31	0.2688 (2)	0.8659 (4)	0.47755 (11)	0.0558 (10)	
H31	0.286239	0.866489	0.456916	0.067*	
C32	0.2939 (2)	0.7789 (4)	0.50326 (11)	0.0564 (10)	
H32	0.328645	0.722834	0.499982	0.068*	
C33	0.26782 (18)	0.7743 (4)	0.53404 (10)	0.0501 (9)	
C34	0.21765 (19)	0.8619 (4)	0.53938 (10)	0.0546 (9)	
H34	0.200294	0.860738	0.560032	0.066*	
C35	0.19397 (19)	0.9501 (4)	0.51382 (11)	0.0564 (10)	
H35	0.161172	1.009884	0.517789	0.068*	
C36	0.1881 (2)	1.0426 (4)	0.45481 (11)	0.0599 (10)	
H36	0.157097	1.102365	0.460922	0.072*	
C37	0.1998 (2)	1.0500 (4)	0.42242 (11)	0.0586 (10)	
H37	0.230420	0.992554	0.414903	0.070*	
C38	0.1654 (2)	1.1472 (4)	0.39823 (11)	0.0648 (11)	
O39	0.1217 (2)	1.2194 (4)	0.40610 (9)	0.1044 (14)	
O40	0.28783 (13)	0.6884 (3)	0.56044 (7)	0.0577 (7)	
C41	0.3425 (2)	0.6041 (4)	0.55772 (11)	0.0571 (10)	
H41A	0.382700	0.656515	0.558958	0.069*	
H41B	0.335574	0.559114	0.535619	0.069*	
C42	0.3503 (2)	0.5029 (4)	0.58653 (11)	0.0557 (10)	
O43	0.39865 (15)	0.4300 (3)	0.58989 (8)	0.0778 (9)	
N44	0.30178 (18)	0.5002 (3)	0.60640 (9)	0.0562 (8)	
H44	0.2711 (16)	0.550 (3)	0.5984 (10)	0.058 (12)*	
C45	0.2971 (2)	0.4147 (4)	0.63466 (10)	0.0532 (9)	
C46	0.3525 (2)	0.3725 (5)	0.65661 (12)	0.0683 (11)	
H46	0.394592	0.395325	0.652004	0.082*	
C47	0.3462 (2)	0.2969 (5)	0.68534 (12)	0.0732 (12)	
H47	0.383861	0.269683	0.699975	0.088*	
C48	0.2845 (2)	0.2617 (4)	0.69238 (11)	0.0659 (12)	
C49	0.2284 (2)	0.3034 (5)	0.67035 (13)	0.0680 (12)	
H49	0.186471	0.280207	0.675023	0.082*	
C50	0.2345 (2)	0.3792 (4)	0.64155 (11)	0.0597 (10)	
H50	0.196850	0.406161	0.626867	0.072*	
O51	0.27261 (19)	0.1882 (4)	0.72034 (9)	0.0913 (11)	
C52	0.3286 (3)	0.1255 (6)	0.74011 (15)	0.116 (2)	
H52C	0.350910	0.071550	0.725238	0.174*	
H52A	0.313871	0.070994	0.757582	0.174*	
H52B	0.358526	0.191849	0.750803	0.174*	
C53	0.18558 (18)	1.1576 (4)	0.36303 (10)	0.0537 (9)	
C54	0.2096 (2)	1.0521 (4)	0.34642 (12)	0.0666 (12)	
H54	0.214911	0.970349	0.357439	0.080*	
C55	0.2258 (3)	1.0657 (5)	0.31364 (14)	0.0847 (15)	
H55	0.241093	0.992980	0.302515	0.102*	
C56	0.2194 (3)	1.1880 (6)	0.29730 (15)	0.0879 (16)	
H56	0.231169	1.198326	0.275402	0.105*	
C57	0.1955 (3)	1.2938 (5)	0.31388 (14)	0.0806 (14)	
H57	0.191220	1.376129	0.303136	0.097*	
C58	0.1778 (2)	1.2789 (4)	0.34613 (12)	0.0692 (12)	
H58	0.160503	1.350535	0.356776	0.083*	
C59	0.51946 (19)	−0.1497 (4)	0.50151 (11)	0.0551 (9)	
C60	0.5433 (2)	−0.1457 (4)	0.47008 (11)	0.0595 (10)	
H60	0.576815	−0.204088	0.466273	0.071*	
C61	0.51886 (19)	−0.0577 (4)	0.44428 (10)	0.0551 (9)	
H61	0.536705	−0.054912	0.423771	0.066*	
C62	0.46741 (18)	0.0262 (4)	0.44940 (10)	0.0498 (9)	
C63	0.4409 (2)	0.0206 (4)	0.48009 (12)	0.0614 (11)	
H63	0.405358	0.074887	0.483238	0.074*	
C64	0.4670 (2)	−0.0653 (4)	0.50581 (11)	0.0576 (10)	
H64	0.449456	−0.067137	0.526405	0.069*	
C65	0.5502 (2)	−0.2382 (4)	0.52870 (12)	0.0611 (11)	
H65	0.582285	−0.295401	0.522506	0.073*	
C66	0.5388 (2)	−0.2481 (4)	0.56086 (11)	0.0626 (11)	
H66	0.506992	−0.193547	0.568359	0.075*	
C67	0.5750 (2)	−0.3434 (4)	0.58526 (12)	0.0677 (12)	
O68	0.6213 (2)	−0.4079 (4)	0.57737 (10)	0.1051 (14)	
O69	0.44651 (14)	0.1113 (3)	0.42267 (7)	0.0626 (7)	
C70	0.3923 (2)	0.1975 (4)	0.42533 (12)	0.0601 (10)	
H70A	0.398817	0.240882	0.447654	0.072*	
H70B	0.351460	0.146815	0.423353	0.072*	
C71	0.3872 (2)	0.3005 (4)	0.39695 (11)	0.0579 (10)	
O72	0.33986 (15)	0.3769 (3)	0.39345 (9)	0.0806 (9)	
N73	0.43728 (18)	0.3014 (3)	0.37827 (9)	0.0602 (9)	
H73	0.469 (2)	0.247 (5)	0.3849 (12)	0.071 (14)*	
C74	0.4471 (2)	0.3866 (4)	0.35075 (10)	0.0539 (9)	
C75	0.5107 (2)	0.4094 (5)	0.34443 (11)	0.0656 (11)	
H75	0.545996	0.374761	0.359556	0.079*	
C76	0.5241 (2)	0.4822 (5)	0.31633 (12)	0.0701 (12)	
H76	0.567623	0.494669	0.312459	0.084*	
C77	0.4723 (2)	0.5358 (4)	0.29421 (11)	0.0624 (11)	
C78	0.4079 (2)	0.5185 (4)	0.30088 (12)	0.0654 (11)	
H78	0.372937	0.557152	0.286339	0.078*	
C79	0.3947 (2)	0.4444 (4)	0.32889 (11)	0.0620 (11)	
H79	0.351291	0.433365	0.333057	0.074*	
O80	0.47978 (19)	0.6065 (4)	0.26502 (9)	0.0856 (10)	
C81	0.5450 (3)	0.6318 (5)	0.25895 (15)	0.1006 (17)	
H81A	0.568257	0.680799	0.277965	0.151*	
H81B	0.543744	0.682508	0.238141	0.151*	
H81C	0.567377	0.549416	0.256585	0.151*	
C82	0.5543 (2)	−0.3619 (4)	0.61985 (11)	0.0583 (10)	
C83	0.5653 (2)	−0.4838 (4)	0.63590 (13)	0.0722 (13)	
H83	0.585934	−0.550590	0.625061	0.087*	
C84	0.5461 (3)	−0.5082 (5)	0.66765 (15)	0.0887 (16)	
H84	0.553008	−0.591133	0.677863	0.106*	
C85	0.5166 (3)	−0.4089 (6)	0.68410 (14)	0.0920 (17)	
H85	0.503552	−0.424451	0.705510	0.110*	
C86	0.5064 (3)	−0.2870 (5)	0.66883 (14)	0.0911 (17)	
H86	0.487663	−0.219444	0.680331	0.109*	
C87	0.5238 (2)	−0.2630 (5)	0.63633 (13)	0.0749 (13)	
H87	0.514999	−0.181081	0.625773	0.090*	

### Atomic displacement parameters (Å^2^)

**Table:**

	*U*^11^	*U*^22^	*U*^33^	*U*^12^	*U*^13^	*U*^23^
C1	0.051 (2)	0.052 (2)	0.055 (2)	0.0038 (17)	0.0080 (18)	−0.0024 (18)
C2	0.056 (2)	0.063 (2)	0.054 (2)	0.0137 (19)	0.0099 (18)	−0.005 (2)
C3	0.058 (2)	0.066 (2)	0.049 (2)	0.0032 (19)	0.0127 (18)	−0.0037 (19)
C4	0.051 (2)	0.052 (2)	0.048 (2)	−0.0030 (17)	0.0084 (17)	0.0011 (17)
C5	0.065 (2)	0.055 (2)	0.062 (3)	0.0143 (19)	0.022 (2)	0.012 (2)
C6	0.061 (2)	0.057 (2)	0.058 (3)	0.0093 (18)	0.0196 (19)	0.0072 (19)
C7	0.061 (2)	0.052 (2)	0.065 (3)	0.0111 (18)	0.012 (2)	0.001 (2)
C8	0.056 (2)	0.057 (2)	0.066 (3)	0.0110 (19)	0.010 (2)	0.007 (2)
C9	0.062 (3)	0.058 (2)	0.065 (3)	0.0145 (19)	0.006 (2)	0.007 (2)
O10	0.081 (2)	0.115 (3)	0.079 (2)	0.047 (2)	0.0240 (18)	0.027 (2)
O11	0.0723 (18)	0.0597 (16)	0.0514 (17)	0.0099 (14)	0.0157 (14)	0.0087 (14)
C12	0.055 (2)	0.056 (2)	0.056 (2)	0.0014 (18)	0.0105 (18)	0.0037 (19)
C13	0.057 (2)	0.055 (2)	0.047 (2)	−0.0007 (18)	0.0048 (18)	−0.0007 (18)
O14	0.0633 (17)	0.0785 (19)	0.064 (2)	0.0156 (15)	0.0160 (14)	0.0136 (16)
N15	0.069 (2)	0.056 (2)	0.050 (2)	0.0109 (17)	0.0123 (18)	0.0059 (16)
C16	0.063 (2)	0.0473 (19)	0.039 (2)	0.0047 (17)	0.0069 (17)	−0.0008 (16)
C17	0.068 (3)	0.059 (2)	0.064 (3)	0.017 (2)	0.016 (2)	0.008 (2)
C18	0.080 (3)	0.068 (3)	0.076 (3)	0.023 (2)	0.035 (3)	0.013 (3)
C19	0.070 (2)	0.052 (2)	0.045 (2)	−0.0002 (19)	0.0103 (19)	−0.0001 (18)
C20	0.064 (2)	0.060 (2)	0.052 (2)	0.0108 (19)	0.0044 (19)	0.0057 (19)
C21	0.060 (2)	0.067 (3)	0.050 (2)	0.0162 (19)	0.0145 (18)	0.008 (2)
O22	0.101 (2)	0.076 (2)	0.064 (2)	0.0140 (18)	0.0300 (18)	0.0195 (18)
C23	0.123 (4)	0.062 (3)	0.066 (3)	0.008 (3)	0.014 (3)	0.014 (2)
C24	0.065 (3)	0.047 (2)	0.057 (3)	0.0096 (18)	0.005 (2)	0.0014 (18)
C25	0.070 (3)	0.071 (3)	0.072 (3)	0.000 (2)	0.010 (2)	0.008 (2)
C26	0.087 (3)	0.077 (3)	0.093 (4)	−0.004 (3)	0.029 (3)	0.003 (3)
C27	0.118 (5)	0.078 (3)	0.067 (3)	0.010 (3)	0.029 (3)	0.003 (3)
C28	0.105 (4)	0.103 (4)	0.057 (3)	0.021 (3)	0.003 (3)	0.003 (3)
C29	0.071 (3)	0.079 (3)	0.065 (3)	0.016 (2)	0.007 (2)	0.002 (2)
C88	0.052 (2)	0.0456 (19)	0.055 (2)	0.0039 (16)	0.0072 (18)	0.0022 (17)
C89	0.062 (2)	0.059 (2)	0.056 (2)	0.0101 (19)	0.0210 (19)	0.0101 (19)
C90	0.063 (2)	0.059 (2)	0.064 (3)	0.0171 (19)	0.020 (2)	0.010 (2)
C91	0.056 (2)	0.049 (2)	0.051 (2)	−0.0007 (17)	0.0073 (18)	0.0009 (17)
C92	0.059 (2)	0.065 (2)	0.049 (2)	0.0057 (19)	0.0100 (18)	−0.0013 (19)
C93	0.056 (2)	0.068 (3)	0.062 (3)	0.0160 (19)	0.010 (2)	−0.007 (2)
C94	0.059 (2)	0.056 (2)	0.066 (3)	0.0123 (19)	0.006 (2)	0.001 (2)
C95	0.058 (2)	0.055 (2)	0.059 (3)	0.0116 (18)	0.003 (2)	0.002 (2)
C96	0.068 (3)	0.056 (2)	0.067 (3)	0.015 (2)	0.006 (2)	0.007 (2)
O97	0.092 (2)	0.114 (3)	0.078 (2)	0.051 (2)	0.0194 (19)	0.026 (2)
O98	0.0754 (18)	0.0605 (16)	0.0518 (17)	0.0133 (14)	0.0152 (14)	0.0099 (14)
C99	0.060 (2)	0.061 (2)	0.058 (3)	0.0073 (19)	0.0086 (19)	0.009 (2)
C100	0.063 (2)	0.053 (2)	0.047 (2)	−0.0003 (18)	0.0053 (19)	0.0008 (18)
O101	0.0686 (18)	0.0690 (18)	0.068 (2)	0.0168 (15)	0.0101 (15)	0.0155 (15)
N102	0.075 (2)	0.0547 (19)	0.054 (2)	0.0148 (18)	0.0113 (18)	0.0093 (17)
C103	0.069 (2)	0.052 (2)	0.042 (2)	0.0030 (18)	0.0049 (18)	−0.0005 (17)
C104	0.088 (3)	0.064 (3)	0.076 (3)	0.024 (2)	0.020 (3)	0.012 (2)
C105	0.102 (4)	0.079 (3)	0.077 (3)	0.025 (3)	0.038 (3)	0.016 (3)
C106	0.082 (3)	0.058 (2)	0.046 (2)	0.004 (2)	0.009 (2)	0.0041 (19)
C107	0.078 (3)	0.058 (2)	0.056 (3)	0.018 (2)	0.007 (2)	0.009 (2)
C108	0.078 (3)	0.065 (3)	0.065 (3)	0.018 (2)	0.018 (2)	0.009 (2)
O109	0.119 (3)	0.080 (2)	0.064 (2)	0.020 (2)	0.033 (2)	0.0176 (18)
C110	0.100 (4)	0.066 (3)	0.070 (3)	−0.002 (3)	0.008 (3)	0.018 (2)
C111	0.063 (2)	0.047 (2)	0.061 (3)	0.0086 (18)	0.009 (2)	0.0050 (19)
C112	0.068 (3)	0.076 (3)	0.081 (4)	0.004 (2)	0.007 (2)	0.015 (3)
C113	0.087 (4)	0.086 (4)	0.111 (5)	0.010 (3)	0.034 (4)	0.022 (3)
C114	0.120 (5)	0.081 (3)	0.081 (4)	0.015 (3)	0.037 (4)	0.021 (3)
C115	0.098 (4)	0.096 (4)	0.065 (3)	0.018 (3)	0.008 (3)	0.005 (3)
C116	0.069 (3)	0.076 (3)	0.068 (3)	0.012 (2)	0.004 (2)	0.004 (2)
C30	0.052 (2)	0.051 (2)	0.046 (2)	0.0023 (16)	0.0106 (17)	0.0015 (17)
C31	0.062 (2)	0.061 (2)	0.049 (2)	0.0089 (18)	0.0214 (18)	0.0063 (19)
C32	0.063 (2)	0.055 (2)	0.054 (2)	0.0127 (18)	0.0212 (19)	0.0092 (19)
C33	0.055 (2)	0.049 (2)	0.047 (2)	−0.0025 (17)	0.0110 (17)	0.0022 (17)
C34	0.059 (2)	0.060 (2)	0.048 (2)	0.0015 (18)	0.0180 (18)	0.0007 (18)
C35	0.054 (2)	0.061 (2)	0.057 (2)	0.0114 (18)	0.0149 (19)	0.001 (2)
C36	0.058 (2)	0.062 (2)	0.060 (3)	0.0105 (19)	0.011 (2)	0.005 (2)
C37	0.064 (2)	0.056 (2)	0.056 (3)	0.0149 (19)	0.0090 (19)	0.0046 (19)
C38	0.078 (3)	0.061 (2)	0.056 (3)	0.021 (2)	0.011 (2)	0.005 (2)
O39	0.130 (3)	0.118 (3)	0.070 (2)	0.074 (3)	0.032 (2)	0.021 (2)
O40	0.0697 (17)	0.0574 (15)	0.0491 (16)	0.0109 (13)	0.0186 (13)	0.0101 (13)
C41	0.061 (2)	0.061 (2)	0.052 (2)	0.0056 (19)	0.0161 (19)	0.0111 (19)
C42	0.062 (2)	0.058 (2)	0.049 (2)	0.0061 (19)	0.0125 (19)	0.0025 (19)
O43	0.0728 (19)	0.095 (2)	0.069 (2)	0.0304 (18)	0.0229 (16)	0.0248 (18)
N44	0.066 (2)	0.0576 (19)	0.046 (2)	0.0138 (17)	0.0113 (17)	0.0105 (16)
C45	0.065 (2)	0.051 (2)	0.044 (2)	0.0126 (18)	0.0098 (18)	0.0024 (18)
C46	0.064 (2)	0.083 (3)	0.058 (3)	0.014 (2)	0.011 (2)	0.015 (2)
C47	0.073 (3)	0.089 (3)	0.057 (3)	0.027 (2)	0.009 (2)	0.023 (2)
C48	0.088 (3)	0.064 (2)	0.047 (2)	0.024 (2)	0.017 (2)	0.015 (2)
C49	0.073 (3)	0.068 (3)	0.067 (3)	0.013 (2)	0.022 (2)	0.011 (2)
C50	0.064 (2)	0.061 (2)	0.054 (2)	0.015 (2)	0.0065 (19)	0.007 (2)
O51	0.114 (3)	0.099 (2)	0.067 (2)	0.033 (2)	0.035 (2)	0.038 (2)
C52	0.143 (5)	0.127 (5)	0.082 (4)	0.055 (4)	0.030 (4)	0.060 (4)
C53	0.053 (2)	0.055 (2)	0.052 (2)	0.0075 (17)	0.0038 (17)	0.0031 (18)
C54	0.076 (3)	0.060 (2)	0.067 (3)	0.019 (2)	0.019 (2)	0.007 (2)
C55	0.100 (4)	0.091 (3)	0.068 (3)	0.031 (3)	0.026 (3)	0.003 (3)
C56	0.086 (3)	0.112 (4)	0.070 (3)	0.016 (3)	0.026 (3)	0.021 (3)
C57	0.089 (3)	0.074 (3)	0.078 (3)	0.002 (3)	0.010 (3)	0.022 (3)
C58	0.084 (3)	0.056 (2)	0.065 (3)	0.005 (2)	0.005 (2)	0.007 (2)
C59	0.057 (2)	0.053 (2)	0.056 (2)	0.0001 (17)	0.0122 (19)	−0.0021 (19)
C60	0.054 (2)	0.067 (3)	0.059 (3)	0.0094 (19)	0.0129 (19)	−0.004 (2)
C61	0.057 (2)	0.062 (2)	0.049 (2)	0.0030 (18)	0.0180 (18)	−0.0036 (19)
C62	0.057 (2)	0.0445 (19)	0.048 (2)	−0.0007 (16)	0.0082 (18)	−0.0013 (17)
C63	0.068 (2)	0.056 (2)	0.064 (3)	0.0151 (19)	0.021 (2)	0.004 (2)
C64	0.065 (2)	0.055 (2)	0.056 (3)	0.0050 (18)	0.020 (2)	0.0077 (19)
C65	0.061 (2)	0.058 (2)	0.066 (3)	0.0106 (19)	0.015 (2)	−0.001 (2)
C66	0.065 (2)	0.067 (3)	0.057 (3)	0.011 (2)	0.015 (2)	0.005 (2)
C67	0.072 (3)	0.070 (3)	0.063 (3)	0.021 (2)	0.014 (2)	0.005 (2)
O68	0.118 (3)	0.124 (3)	0.082 (3)	0.072 (3)	0.041 (2)	0.028 (2)
O69	0.0748 (18)	0.0579 (16)	0.0579 (17)	0.0116 (14)	0.0196 (14)	0.0082 (13)
C70	0.061 (2)	0.056 (2)	0.065 (3)	0.0062 (19)	0.015 (2)	0.005 (2)
C71	0.060 (2)	0.054 (2)	0.058 (3)	0.0060 (19)	0.004 (2)	0.0012 (19)
O72	0.0680 (19)	0.090 (2)	0.085 (2)	0.0241 (17)	0.0156 (17)	0.0182 (19)
N73	0.068 (2)	0.0568 (19)	0.056 (2)	0.0148 (17)	0.0104 (18)	0.0085 (17)
C74	0.063 (2)	0.050 (2)	0.047 (2)	0.0104 (18)	0.0020 (18)	0.0008 (17)
C75	0.065 (3)	0.077 (3)	0.053 (2)	0.014 (2)	0.004 (2)	0.010 (2)
C76	0.070 (3)	0.081 (3)	0.058 (3)	0.001 (2)	0.007 (2)	0.006 (2)
C77	0.083 (3)	0.055 (2)	0.047 (2)	−0.001 (2)	0.002 (2)	0.0060 (19)
C78	0.070 (3)	0.063 (2)	0.057 (3)	0.010 (2)	−0.011 (2)	0.005 (2)
C79	0.061 (2)	0.064 (2)	0.058 (3)	0.0044 (19)	0.002 (2)	−0.002 (2)
O80	0.108 (3)	0.085 (2)	0.062 (2)	−0.0071 (19)	0.0049 (19)	0.0176 (18)
C81	0.136 (5)	0.092 (4)	0.080 (4)	−0.013 (4)	0.037 (4)	0.010 (3)
C82	0.062 (2)	0.057 (2)	0.056 (2)	0.0113 (18)	0.0075 (19)	−0.0008 (19)
C83	0.094 (3)	0.054 (2)	0.069 (3)	0.022 (2)	0.015 (3)	0.003 (2)
C84	0.118 (4)	0.068 (3)	0.086 (4)	0.024 (3)	0.030 (3)	0.019 (3)
C85	0.118 (4)	0.096 (4)	0.068 (3)	0.032 (3)	0.034 (3)	0.022 (3)
C86	0.130 (5)	0.079 (3)	0.069 (3)	0.042 (3)	0.032 (3)	0.005 (3)
C87	0.096 (3)	0.065 (3)	0.066 (3)	0.020 (2)	0.018 (3)	0.009 (2)

### Geometric parameters (Å, º)

**Table:**

C1—C2	1.391 (6)	C30—C31	1.399 (5)
C1—C6	1.396 (5)	C30—C35	1.400 (5)
C1—C7	1.462 (6)	C30—C36	1.456 (5)
C2—H2	0.9300	C31—H31	0.9300
C2—C3	1.381 (6)	C31—C32	1.377 (5)
C3—H3	0.9300	C32—H32	0.9300
C3—C4	1.379 (5)	C32—C33	1.389 (5)
C4—C5	1.391 (5)	C33—C34	1.390 (5)
C4—O11	1.368 (4)	C33—O40	1.366 (4)
C5—H5	0.9300	C34—H34	0.9300
C5—C6	1.382 (6)	C34—C35	1.374 (5)
C6—H6	0.9300	C35—H35	0.9300
C7—H7	0.9300	C36—H36	0.9300
C7—C8	1.316 (6)	C36—C37	1.328 (6)
C8—H8	0.9300	C37—H37	0.9300
C8—C9	1.471 (6)	C37—C38	1.470 (6)
C9—O10	1.230 (5)	C38—O39	1.223 (5)
C9—C24	1.491 (6)	C38—C53	1.501 (6)
O11—C12	1.425 (4)	O40—C41	1.418 (5)
C12—H12A	0.9700	C41—H41A	0.9700
C12—H12B	0.9700	C41—H41B	0.9700
C12—C13	1.510 (5)	C41—C42	1.514 (5)
C13—O14	1.230 (5)	C42—O43	1.221 (5)
C13—N15	1.330 (5)	C42—N44	1.345 (5)
N15—H15	0.85 (5)	N44—H44	0.83 (2)
N15—C16	1.419 (5)	N44—C45	1.419 (5)
C16—C17	1.378 (5)	C45—C46	1.383 (6)
C16—C21	1.390 (5)	C45—C50	1.388 (6)
C17—H17	0.9300	C46—H46	0.9300
C17—C18	1.369 (6)	C46—C47	1.382 (6)
C18—H18	0.9300	C47—H47	0.9300
C18—C19	1.381 (6)	C47—C48	1.372 (7)
C19—C20	1.372 (6)	C48—C49	1.391 (6)
C19—O22	1.382 (5)	C48—O51	1.375 (5)
C20—H20	0.9300	C49—H49	0.9300
C20—C21	1.388 (6)	C49—C50	1.384 (6)
C21—H21	0.9300	C50—H50	0.9300
O22—C23	1.414 (5)	O51—C52	1.429 (6)
C23—H23A	0.9600	C52—H52C	0.9600
C23—H23B	0.9600	C52—H52A	0.9600
C23—H23C	0.9600	C52—H52B	0.9600
C24—C25	1.393 (6)	C53—C54	1.375 (6)
C24—C29	1.392 (6)	C53—C58	1.390 (6)
C25—H25	0.9300	C54—H54	0.9300
C25—C26	1.380 (7)	C54—C55	1.380 (6)
C26—H26	0.9300	C55—H55	0.9300
C26—C27	1.363 (8)	C55—C56	1.389 (7)
C27—H27	0.9300	C56—H56	0.9300
C27—C28	1.377 (8)	C56—C57	1.376 (7)
C28—H28	0.9300	C57—H57	0.9300
C28—C29	1.386 (7)	C57—C58	1.373 (7)
C29—H29	0.9300	C58—H58	0.9300
C88—C89	1.391 (5)	C59—C60	1.390 (6)
C88—C93	1.399 (6)	C59—C64	1.396 (5)
C88—C94	1.461 (5)	C59—C65	1.460 (6)
C89—H89	0.9300	C60—H60	0.9300
C89—C90	1.382 (5)	C60—C61	1.383 (6)
C90—H90	0.9300	C61—H61	0.9300
C90—C91	1.387 (5)	C61—C62	1.385 (5)
C91—C92	1.386 (5)	C62—C63	1.391 (6)
C91—O98	1.367 (4)	C62—O69	1.374 (5)
C92—H92	0.9300	C63—H63	0.9300
C92—C93	1.376 (6)	C63—C64	1.376 (6)
C93—H93	0.9300	C64—H64	0.9300
C94—H94	0.9300	C65—H65	0.9300
C94—C95	1.327 (6)	C65—C66	1.319 (6)
C95—H95	0.9300	C66—H66	0.9300
C95—C96	1.471 (6)	C66—C67	1.475 (6)
C96—O97	1.238 (5)	C67—O68	1.223 (5)
C96—C111	1.506 (6)	C67—C82	1.490 (6)
O98—C99	1.421 (5)	O69—C70	1.422 (5)
C99—H99A	0.9700	C70—H70A	0.9700
C99—H99B	0.9700	C70—H70B	0.9700
C99—C100	1.519 (5)	C70—C71	1.516 (6)
C100—O101	1.219 (5)	C71—O72	1.226 (5)
C100—N102	1.343 (5)	C71—N73	1.340 (5)
N102—H102	0.86 (4)	N73—H73	0.86 (4)
N102—C103	1.411 (5)	N73—C74	1.417 (5)
C103—C104	1.385 (6)	C74—C75	1.375 (6)
C103—C108	1.382 (6)	C74—C79	1.394 (5)
C104—H104	0.9300	C75—H75	0.9300
C104—C105	1.376 (6)	C75—C76	1.384 (6)
C105—H105	0.9300	C76—H76	0.9300
C105—C106	1.387 (6)	C76—C77	1.375 (6)
C106—C107	1.360 (6)	C77—C78	1.386 (6)
C106—O109	1.379 (5)	C77—O80	1.376 (5)
C107—H107	0.9300	C78—H78	0.9300
C107—C108	1.390 (6)	C78—C79	1.387 (6)
C108—H108	0.9300	C79—H79	0.9300
O109—C110	1.403 (5)	O80—C81	1.406 (6)
C110—H11A	0.9600	C81—H81A	0.9600
C110—H11B	0.9600	C81—H81B	0.9600
C110—H11C	0.9600	C81—H81C	0.9600
C111—C112	1.367 (6)	C82—C83	1.385 (6)
C111—C116	1.386 (6)	C82—C87	1.386 (6)
C112—H112	0.9300	C83—H83	0.9300
C112—C113	1.381 (7)	C83—C84	1.380 (7)
C113—H113	0.9300	C84—H84	0.9300
C113—C114	1.386 (8)	C84—C85	1.377 (7)
C114—H114	0.9300	C85—H85	0.9300
C114—C115	1.373 (7)	C85—C86	1.370 (7)
C115—H115	0.9300	C86—H86	0.9300
C115—C116	1.381 (7)	C86—C87	1.393 (7)
C116—H116	0.9300	C87—H87	0.9300
			
C2—C1—C6	116.9 (4)	C31—C30—C35	117.2 (3)
C2—C1—C7	120.4 (3)	C31—C30—C36	122.2 (4)
C6—C1—C7	122.7 (4)	C35—C30—C36	120.6 (3)
C1—C2—H2	118.7	C30—C31—H31	119.5
C3—C2—C1	122.5 (4)	C32—C31—C30	121.0 (4)
C3—C2—H2	118.7	C32—C31—H31	119.5
C2—C3—H3	120.3	C31—C32—H32	119.7
C4—C3—C2	119.5 (4)	C31—C32—C33	120.6 (4)
C4—C3—H3	120.3	C33—C32—H32	119.7
C3—C4—C5	119.5 (4)	C32—C33—C34	119.5 (4)
O11—C4—C3	116.0 (3)	O40—C33—C32	124.8 (3)
O11—C4—C5	124.5 (3)	O40—C33—C34	115.7 (3)
C4—C5—H5	119.9	C33—C34—H34	120.3
C6—C5—C4	120.3 (4)	C35—C34—C33	119.3 (4)
C6—C5—H5	119.9	C35—C34—H34	120.3
C1—C6—H6	119.3	C30—C35—H35	118.8
C5—C6—C1	121.3 (4)	C34—C35—C30	122.3 (4)
C5—C6—H6	119.3	C34—C35—H35	118.8
C1—C7—H7	115.3	C30—C36—H36	115.7
C8—C7—C1	129.5 (4)	C37—C36—C30	128.5 (4)
C8—C7—H7	115.3	C37—C36—H36	115.7
C7—C8—H8	119.0	C36—C37—H37	119.4
C7—C8—C9	122.0 (4)	C36—C37—C38	121.2 (4)
C9—C8—H8	119.0	C38—C37—H37	119.4
C8—C9—C24	119.3 (4)	C37—C38—C53	118.0 (4)
O10—C9—C8	120.9 (4)	O39—C38—C37	122.1 (4)
O10—C9—C24	119.8 (4)	O39—C38—C53	119.9 (4)
C4—O11—C12	118.2 (3)	C33—O40—C41	118.3 (3)
O11—C12—H12A	109.5	O40—C41—H41A	109.6
O11—C12—H12B	109.5	O40—C41—H41B	109.6
O11—C12—C13	110.6 (3)	O40—C41—C42	110.3 (3)
H12A—C12—H12B	108.1	H41A—C41—H41B	108.1
C13—C12—H12A	109.5	C42—C41—H41A	109.6
C13—C12—H12B	109.5	C42—C41—H41B	109.6
O14—C13—C12	118.1 (4)	O43—C42—C41	118.8 (4)
O14—C13—N15	125.9 (4)	O43—C42—N44	125.1 (4)
N15—C13—C12	116.0 (4)	N44—C42—C41	116.1 (3)
C13—N15—H15	115 (4)	C42—N44—H44	110 (3)
C13—N15—C16	129.2 (4)	C42—N44—C45	127.1 (3)
C16—N15—H15	114 (4)	C45—N44—H44	122 (3)
C17—C16—N15	118.0 (4)	C46—C45—N44	122.1 (4)
C17—C16—C21	118.1 (4)	C46—C45—C50	119.3 (4)
C21—C16—N15	123.9 (4)	C50—C45—N44	118.6 (3)
C16—C17—H17	119.4	C45—C46—H46	119.6
C18—C17—C16	121.2 (4)	C45—C46—C47	120.7 (4)
C18—C17—H17	119.4	C47—C46—H46	119.6
C17—C18—H18	119.6	C46—C47—H47	119.8
C17—C18—C19	120.8 (4)	C48—C47—C46	120.3 (4)
C19—C18—H18	119.6	C48—C47—H47	119.8
C18—C19—O22	116.1 (4)	C47—C48—C49	119.3 (4)
C20—C19—C18	119.1 (4)	C47—C48—O51	125.0 (4)
C20—C19—O22	124.8 (4)	O51—C48—C49	115.7 (4)
C19—C20—H20	119.9	C48—C49—H49	119.7
C19—C20—C21	120.1 (4)	C50—C49—C48	120.6 (4)
C21—C20—H20	119.9	C50—C49—H49	119.7
C16—C21—H21	119.6	C45—C50—H50	120.1
C20—C21—C16	120.8 (4)	C49—C50—C45	119.8 (4)
C20—C21—H21	119.6	C49—C50—H50	120.1
C19—O22—C23	117.4 (4)	C48—O51—C52	116.9 (4)
O22—C23—H23A	109.5	O51—C52—H52C	109.5
O22—C23—H23B	109.5	O51—C52—H52A	109.5
O22—C23—H23C	109.5	O51—C52—H52B	109.5
H23A—C23—H23B	109.5	H52C—C52—H52A	109.5
H23A—C23—H23C	109.5	H52C—C52—H52B	109.5
H23B—C23—H23C	109.5	H52A—C52—H52B	109.5
C25—C24—C9	121.4 (4)	C54—C53—C38	123.1 (4)
C29—C24—C9	120.2 (4)	C54—C53—C58	118.7 (4)
C29—C24—C25	118.4 (4)	C58—C53—C38	118.2 (4)
C24—C25—H25	120.3	C53—C54—H54	119.5
C26—C25—C24	119.4 (5)	C53—C54—C55	120.9 (4)
C26—C25—H25	120.3	C55—C54—H54	119.5
C25—C26—H26	119.0	C54—C55—H55	120.0
C27—C26—C25	122.0 (5)	C54—C55—C56	119.9 (5)
C27—C26—H26	119.0	C56—C55—H55	120.0
C26—C27—H27	120.4	C55—C56—H56	120.4
C26—C27—C28	119.3 (5)	C57—C56—C55	119.2 (5)
C28—C27—H27	120.4	C57—C56—H56	120.4
C27—C28—H28	120.1	C56—C57—H57	119.7
C27—C28—C29	119.9 (5)	C58—C57—C56	120.6 (5)
C29—C28—H28	120.1	C58—C57—H57	119.7
C24—C29—H29	119.5	C53—C58—H58	119.7
C28—C29—C24	121.0 (5)	C57—C58—C53	120.6 (4)
C28—C29—H29	119.5	C57—C58—H58	119.7
C89—C88—C93	117.1 (4)	C60—C59—C64	117.6 (4)
C89—C88—C94	123.1 (4)	C60—C59—C65	119.7 (3)
C93—C88—C94	119.7 (3)	C64—C59—C65	122.7 (4)
C88—C89—H89	119.5	C59—C60—H60	118.9
C90—C89—C88	121.1 (4)	C61—C60—C59	122.1 (4)
C90—C89—H89	119.5	C61—C60—H60	118.9
C89—C90—H90	119.7	C60—C61—H61	120.5
C89—C90—C91	120.6 (4)	C60—C61—C62	119.1 (4)
C91—C90—H90	119.7	C62—C61—H61	120.5
C92—C91—C90	119.4 (4)	C61—C62—C63	119.9 (4)
O98—C91—C90	124.9 (3)	O69—C62—C61	115.4 (3)
O98—C91—C92	115.7 (3)	O69—C62—C63	124.7 (3)
C91—C92—H92	120.3	C62—C63—H63	120.0
C93—C92—C91	119.4 (4)	C64—C63—C62	120.1 (4)
C93—C92—H92	120.3	C64—C63—H63	120.0
C88—C93—H93	118.8	C59—C64—H64	119.4
C92—C93—C88	122.4 (4)	C63—C64—C59	121.1 (4)
C92—C93—H93	118.8	C63—C64—H64	119.4
C88—C94—H94	115.6	C59—C65—H65	115.6
C95—C94—C88	128.8 (4)	C66—C65—C59	128.8 (4)
C95—C94—H94	115.6	C66—C65—H65	115.6
C94—C95—H95	119.6	C65—C66—H66	119.1
C94—C95—C96	120.9 (4)	C65—C66—C67	121.8 (4)
C96—C95—H95	119.6	C67—C66—H66	119.1
C95—C96—C111	118.8 (4)	C66—C67—C82	119.4 (4)
O97—C96—C95	121.5 (4)	O68—C67—C66	120.9 (4)
O97—C96—C111	119.7 (4)	O68—C67—C82	119.7 (4)
C91—O98—C99	118.1 (3)	C62—O69—C70	118.9 (3)
O98—C99—H99A	109.5	O69—C70—H70A	109.7
O98—C99—H99B	109.5	O69—C70—H70B	109.7
O98—C99—C100	110.7 (3)	O69—C70—C71	109.6 (3)
H99A—C99—H99B	108.1	H70A—C70—H70B	108.2
C100—C99—H99A	109.5	C71—C70—H70A	109.7
C100—C99—H99B	109.5	C71—C70—H70B	109.7
O101—C100—C99	118.5 (4)	O72—C71—C70	118.8 (4)
O101—C100—N102	125.9 (4)	O72—C71—N73	125.7 (4)
N102—C100—C99	115.6 (4)	N73—C71—C70	115.5 (3)
C100—N102—H102	112 (3)	C71—N73—H73	116 (3)
C100—N102—C103	129.1 (4)	C71—N73—C74	128.6 (4)
C103—N102—H102	118 (3)	C74—N73—H73	116 (3)
C104—C103—N102	117.7 (4)	C75—C74—N73	118.7 (3)
C108—C103—N102	124.4 (4)	C75—C74—C79	118.4 (4)
C108—C103—C104	117.9 (4)	C79—C74—N73	122.8 (4)
C103—C104—H104	119.3	C74—C75—H75	118.9
C105—C104—C103	121.4 (4)	C74—C75—C76	122.2 (4)
C105—C104—H104	119.3	C76—C75—H75	118.9
C104—C105—H105	120.1	C75—C76—H76	120.4
C104—C105—C106	119.8 (5)	C77—C76—C75	119.3 (4)
C106—C105—H105	120.1	C77—C76—H76	120.4
C107—C106—C105	119.4 (4)	C76—C77—C78	119.4 (4)
C107—C106—O109	125.6 (4)	C76—C77—O80	124.1 (4)
O109—C106—C105	115.0 (4)	O80—C77—C78	116.5 (4)
C106—C107—H107	119.6	C77—C78—H78	119.5
C106—C107—C108	120.7 (4)	C77—C78—C79	121.0 (4)
C108—C107—H107	119.6	C79—C78—H78	119.5
C103—C108—C107	120.6 (4)	C74—C79—H79	120.2
C103—C108—H108	119.7	C78—C79—C74	119.6 (4)
C107—C108—H108	119.7	C78—C79—H79	120.2
C106—O109—C110	118.1 (4)	C77—O80—C81	117.2 (4)
O109—C110—H11A	109.5	O80—C81—H81A	109.5
O109—C110—H11B	109.5	O80—C81—H81B	109.5
O109—C110—H11C	109.5	O80—C81—H81C	109.5
H11A—C110—H11B	109.5	H81A—C81—H81B	109.5
H11A—C110—H11C	109.5	H81A—C81—H81C	109.5
H11B—C110—H11C	109.5	H81B—C81—H81C	109.5
C112—C111—C96	121.9 (4)	C83—C82—C67	118.5 (4)
C112—C111—C116	119.3 (4)	C83—C82—C87	118.8 (4)
C116—C111—C96	118.8 (4)	C87—C82—C67	122.7 (4)
C111—C112—H112	119.6	C82—C83—H83	119.3
C111—C112—C113	120.7 (5)	C84—C83—C82	121.3 (4)
C113—C112—H112	119.6	C84—C83—H83	119.3
C112—C113—H113	120.0	C83—C84—H84	120.2
C112—C113—C114	120.0 (5)	C85—C84—C83	119.6 (5)
C114—C113—H113	120.0	C85—C84—H84	120.2
C113—C114—H114	120.3	C84—C85—H85	120.1
C115—C114—C113	119.4 (5)	C86—C85—C84	119.8 (5)
C115—C114—H114	120.3	C86—C85—H85	120.1
C114—C115—H115	119.8	C85—C86—H86	119.5
C114—C115—C116	120.4 (5)	C85—C86—C87	120.9 (5)
C116—C115—H115	119.8	C87—C86—H86	119.5
C111—C116—H116	119.9	C82—C87—C86	119.5 (4)
C115—C116—C111	120.2 (5)	C82—C87—H87	120.2
C115—C116—H116	119.9	C86—C87—H87	120.2
			
C1—C2—C3—C4	−1.7 (6)	C30—C31—C32—C33	1.3 (6)
C1—C7—C8—C9	−173.2 (4)	C30—C36—C37—C38	180.0 (4)
C2—C1—C6—C5	−0.8 (6)	C31—C30—C35—C34	−2.7 (6)
C2—C1—C7—C8	175.4 (4)	C31—C30—C36—C37	5.5 (7)
C2—C3—C4—C5	−0.5 (6)	C31—C32—C33—C34	−2.5 (6)
C2—C3—C4—O11	179.0 (3)	C31—C32—C33—O40	177.6 (4)
C3—C4—C5—C6	2.0 (6)	C32—C33—C34—C35	1.1 (6)
C3—C4—O11—C12	177.8 (3)	C32—C33—O40—C41	5.0 (5)
C4—C5—C6—C1	−1.3 (6)	C33—C34—C35—C30	1.6 (6)
C4—O11—C12—C13	172.3 (3)	C33—O40—C41—C42	−171.3 (3)
C5—C4—O11—C12	−2.7 (6)	C34—C33—O40—C41	−174.9 (3)
C6—C1—C2—C3	2.3 (6)	C35—C30—C31—C32	1.3 (6)
C6—C1—C7—C8	−0.7 (7)	C35—C30—C36—C37	−173.2 (4)
C7—C1—C2—C3	−173.9 (4)	C36—C30—C31—C32	−177.5 (4)
C7—C1—C6—C5	175.4 (4)	C36—C30—C35—C34	176.1 (4)
C7—C8—C9—O10	10.1 (7)	C36—C37—C38—O39	−4.6 (7)
C7—C8—C9—C24	−171.9 (4)	C36—C37—C38—C53	174.7 (4)
C8—C9—C24—C25	30.9 (6)	C37—C38—C53—C54	30.4 (6)
C8—C9—C24—C29	−151.9 (4)	C37—C38—C53—C58	−151.5 (4)
C9—C24—C25—C26	174.1 (4)	C38—C53—C54—C55	178.2 (5)
C9—C24—C29—C28	−175.7 (4)	C38—C53—C58—C57	−179.9 (4)
O10—C9—C24—C25	−151.0 (5)	O39—C38—C53—C54	−150.3 (5)
O10—C9—C24—C29	26.1 (6)	O39—C38—C53—C58	27.8 (7)
O11—C4—C5—C6	−177.5 (4)	O40—C33—C34—C35	−179.0 (3)
O11—C12—C13—O14	177.5 (3)	O40—C41—C42—O43	−173.5 (4)
O11—C12—C13—N15	−3.6 (5)	O40—C41—C42—N44	7.3 (5)
C12—C13—N15—C16	−173.1 (4)	C41—C42—N44—C45	179.5 (4)
C13—N15—C16—C17	174.5 (4)	C42—N44—C45—C46	34.6 (6)
C13—N15—C16—C21	−6.1 (7)	C42—N44—C45—C50	−149.4 (4)
O14—C13—N15—C16	5.6 (7)	O43—C42—N44—C45	0.2 (7)
N15—C16—C17—C18	179.0 (4)	N44—C45—C46—C47	175.4 (4)
N15—C16—C21—C20	−179.4 (4)	N44—C45—C50—C49	−175.5 (4)
C16—C17—C18—C19	−0.1 (8)	C45—C46—C47—C48	0.4 (7)
C17—C16—C21—C20	0.0 (6)	C46—C45—C50—C49	0.6 (7)
C17—C18—C19—C20	1.1 (7)	C46—C47—C48—C49	−0.2 (7)
C17—C18—C19—O22	−178.5 (4)	C46—C47—C48—O51	−179.3 (5)
C18—C19—C20—C21	−1.6 (6)	C47—C48—C49—C50	0.2 (7)
C18—C19—O22—C23	−175.3 (4)	C47—C48—O51—C52	−11.1 (7)
C19—C20—C21—C16	1.0 (6)	C48—C49—C50—C45	−0.4 (7)
C20—C19—O22—C23	5.1 (6)	C49—C48—O51—C52	169.8 (5)
C21—C16—C17—C18	−0.5 (7)	C50—C45—C46—C47	−0.6 (7)
O22—C19—C20—C21	178.0 (4)	O51—C48—C49—C50	179.4 (4)
C24—C25—C26—C27	2.7 (8)	C53—C54—C55—C56	1.4 (8)
C25—C24—C29—C28	1.5 (7)	C54—C53—C58—C57	−1.8 (7)
C25—C26—C27—C28	−0.5 (8)	C54—C55—C56—C57	−1.3 (9)
C26—C27—C28—C29	−1.2 (8)	C55—C56—C57—C58	−0.3 (8)
C27—C28—C29—C24	0.7 (8)	C56—C57—C58—C53	1.8 (8)
C29—C24—C25—C26	−3.1 (7)	C58—C53—C54—C55	0.1 (7)
C88—C89—C90—C91	0.7 (6)	C59—C60—C61—C62	−2.2 (6)
C88—C94—C95—C96	172.1 (4)	C59—C65—C66—C67	−179.6 (4)
C89—C88—C93—C92	−2.3 (6)	C60—C59—C64—C63	−1.3 (6)
C89—C88—C94—C95	2.7 (7)	C60—C59—C65—C66	173.6 (4)
C89—C90—C91—C92	−1.7 (6)	C60—C61—C62—C63	−0.4 (6)
C89—C90—C91—O98	177.9 (4)	C60—C61—C62—O69	179.4 (3)
C90—C91—C92—C93	0.7 (6)	C61—C62—C63—C64	2.1 (6)
C90—C91—O98—C99	0.6 (6)	C61—C62—O69—C70	177.7 (3)
C91—C92—C93—C88	1.4 (6)	C62—C63—C64—C59	−1.2 (6)
C91—O98—C99—C100	−170.4 (3)	C62—O69—C70—C71	168.3 (3)
C92—C91—O98—C99	−179.8 (3)	C63—C62—O69—C70	−2.5 (6)
C93—C88—C89—C90	1.3 (6)	C64—C59—C60—C61	3.0 (6)
C93—C88—C94—C95	−173.7 (4)	C64—C59—C65—C66	−5.1 (7)
C94—C88—C89—C90	−175.2 (4)	C65—C59—C60—C61	−175.8 (4)
C94—C88—C93—C92	174.3 (4)	C65—C59—C64—C63	177.5 (4)
C94—C95—C96—O97	−6.8 (7)	C65—C66—C67—O68	6.9 (8)
C94—C95—C96—C111	175.3 (4)	C65—C66—C67—C82	−172.5 (4)
C95—C96—C111—C112	−32.9 (6)	C66—C67—C82—C83	151.2 (4)
C95—C96—C111—C116	149.0 (4)	C66—C67—C82—C87	−27.8 (7)
C96—C111—C112—C113	−175.7 (4)	C67—C82—C83—C84	−178.7 (5)
C96—C111—C116—C115	178.5 (4)	C67—C82—C87—C86	−179.4 (5)
O97—C96—C111—C112	149.1 (5)	O68—C67—C82—C83	−28.2 (7)
O97—C96—C111—C116	−29.0 (6)	O68—C67—C82—C87	152.8 (5)
O98—C91—C92—C93	−178.9 (4)	O69—C62—C63—C64	−177.7 (4)
O98—C99—C100—O101	−177.7 (4)	O69—C70—C71—O72	174.1 (4)
O98—C99—C100—N102	1.7 (5)	O69—C70—C71—N73	−7.7 (5)
C99—C100—N102—C103	175.1 (4)	C70—C71—N73—C74	−178.7 (4)
C100—N102—C103—C104	−176.3 (4)	C71—N73—C74—C75	153.5 (4)
C100—N102—C103—C108	3.3 (7)	C71—N73—C74—C79	−29.1 (6)
O101—C100—N102—C103	−5.6 (7)	O72—C71—N73—C74	−0.6 (7)
N102—C103—C104—C105	−178.7 (5)	N73—C74—C75—C76	174.4 (4)
N102—C103—C108—C107	179.3 (4)	N73—C74—C79—C78	−175.0 (4)
C103—C104—C105—C106	−0.8 (8)	C74—C75—C76—C77	1.2 (7)
C104—C103—C108—C107	−1.1 (7)	C75—C74—C79—C78	2.4 (6)
C104—C105—C106—C107	−0.6 (8)	C75—C76—C77—C78	1.4 (7)
C104—C105—C106—O109	179.3 (5)	C75—C76—C77—O80	−178.1 (4)
C105—C106—C107—C108	1.1 (7)	C76—C77—C78—C79	−2.1 (7)
C105—C106—O109—C110	168.8 (5)	C76—C77—O80—C81	−4.1 (7)
C106—C107—C108—C103	−0.3 (7)	C77—C78—C79—C74	0.1 (6)
C107—C106—O109—C110	−11.4 (7)	C78—C77—O80—C81	176.3 (4)
C108—C103—C104—C105	1.6 (7)	C79—C74—C75—C76	−3.1 (7)
O109—C106—C107—C108	−178.7 (4)	O80—C77—C78—C79	177.5 (4)
C111—C112—C113—C114	−2.6 (8)	C82—C83—C84—C85	−1.2 (9)
C112—C111—C116—C115	0.4 (7)	C83—C82—C87—C86	1.6 (7)
C112—C113—C114—C115	0.2 (8)	C83—C84—C85—C86	0.1 (10)
C113—C114—C115—C116	2.4 (8)	C84—C85—C86—C87	1.9 (10)
C114—C115—C116—C111	−2.7 (8)	C85—C86—C87—C82	−2.7 (9)
C116—C111—C112—C113	2.3 (7)	C87—C82—C83—C84	0.4 (7)

### Hydrogen-bond geometry (Å, º)

*Cg*1, *Cg*4, *Cg*5, *Cg*7, *Cg*8 and *Cg*10 are the centroids of the C1–C6, C30–C35, C45–C50, C59–C64, C74–C79 and C88–C89 rings, respectively.

**Table:**

*D*—H···*A*	*D*—H	H···*A*	*D*···*A*	*D*—H···*A*
N15—H15···O11	0.84 (5)	2.14 (5)	2.602 (4)	115 (4)
N44—H44···O40	0.83 (3)	2.11 (3)	2.605 (4)	118 (3)
N73—H73···O69	0.86 (4)	2.12 (4)	2.579 (4)	113 (3)
N102—H102···O98	0.86 (4)	2.09 (4)	2.603 (4)	118 (3)
C7—H7···O10	0.93	2.47	2.807 (5)	101
C21—H21···O14	0.93	2.32	2.925 (5)	122
C36—H36···O39	0.93	2.47	2.811 (6)	102
C46—H46···O43	0.93	2.47	2.964 (5)	113
C65—H65···O68	0.93	2.46	2.803 (6)	102
C79—H79···O72	0.93	2.48	2.992 (5)	115
C94—H94···O97	0.93	2.46	2.804 (6)	102
C108—H108···O101	0.93	2.33	2.934 (5)	122
C31—H31···O14	0.93	2.57	3.494 (5)	175
C56—H56···O109^i^	0.93	2.57	3.487 (6)	167
C75—H75···O97^ii^	0.93	2.57	3.409 (6)	151
C114—H114···O51^iii^	0.93	2.56	3.444 (7)	159
C12—H12*A*···*Cg*7^iv^	0.97	2.81	3.522 (4)	131
C23—H23*A*···*Cg*8^iv^	0.96	2.72	3.669 (5)	168
C41—H41*B*···*Cg*10^v^	0.97	2.84	3.592 (4)	135
C70—H70*A*···*Cg*1	0.97	2.92	3.667 (5)	135
C99—H99*B*···*Cg*4^vi^	0.97	2.80	3.517 (4)	131
C110—H11*C*···*Cg*5^vi^	0.96	2.64	3.569 (5)	162

Symmetry codes: (i) *x*−1/2, −*y*+3/2, *z*−1/2; (ii) *x*, *y*−1, *z*; (iii) *x*+1/2, −*y*+3/2, *z*−1/2; (iv) *x*, *y*+1, *z*; (v) *x*−1/2, *y*−1/2, *z*; (vi) *x*+1/2, *y*−1/2, *z*.
